# Long-term effects of prenatal undernutrition on female rat hypothalamic KNDy neurons

**DOI:** 10.1530/EC-22-0307

**Published:** 2022-12-15

**Authors:** Shiori Minabe, Kinuyo Iwata, Youki Watanabe, Hirotaka Ishii, Hitoshi Ozawa

**Affiliations:** 1 Department of Anatomy and Neurobiology, Graduate School of Medicine, Nippon Medical School, Tokyo, Japan; 2 Division of Biomedical Information Analysis, Iwate Tohoku Medical Megabank Organization, Disaster Reconstruction Center, Iwate Medical University, Yahaba, Japan; 3 Faculty of Health Science, Bukkyo University, Kyoto, Japan

**Keywords:** low birth weight, fetal programing, kisspeptin, prenatal undernutrition

## Abstract

The nutritional environment during development periods induces metabolic programming, leading to metabolic disorders and detrimental influences on human reproductive health. This study aimed to determine the long-term adverse effect of intrauterine malnutrition on the reproductive center kisspeptin-neurokinin B-dynorphin A (KNDy) neurons in the hypothalamic arcuate nucleus (ARC) of female offspring. Twelve pregnant rats were divided into *ad-lib-*fed (control, *n*  = 6) and 50% undernutrition (UN, *n*  = 6) groups. The UN group was restricted to 50% daily food intake of the control dams from gestation day 9 until term delivery. Differences between the two groups in terms of various maternal parameters, including body weight (BW), pregnancy duration, and litter size, as well as birth weight, puberty onset, estrous cyclicity, pulsatile luteinizing hormone (LH) secretion, and hypothalamic gene expression of offspring, were determined. Female offspring of UN dams exhibited low BW from birth to 3 weeks, whereas UN offspring showed signs of precocious puberty; hypothalamic *Tac3* (a neurokinin B gene) expression was increased in prepubertal UN offspring, and the BW at the virginal opening was lower in UN offspring than that in the control group. Interestingly, the UN offspring showed significant decreases in the number of KNDy gene-expressing cells after 29 weeks of age, but the number of ARC kisspeptin-immunoreactive cells, pulsatile LH secretions, and estrous cyclicity were comparable between the groups. In conclusion, intrauterine undernutrition induced various changes in KNDy gene expression depending on the life stage. Thus, intrauterine undernutrition affected hypothalamic developmental programming in female rats.

## Introduction

Maternal nutrition during pregnancy and lactation profoundly affects the intrauterine environment, leading to developmental programming in various organs and systems in the offspring (1). This concept, called the developmental origins of health and disease (DOHaD) hypothesis, has been linked to the pathogenesis of metabolic disorders, such as cardiovascular functions (2), kidney functions (3), metabolic syndrome (4), and insulin resistance (5), and obstetrics and gynecology diseases, such as puberty and menstrual disorders, as well as polycystic ovarian syndrome (6). Besides human clinical studies, DOHaD-related reproductive changes have been shown in the offspring of ewes (7, 8, 9, 10), cows (11), mice (12), and rats (13). Maternal nutrient restriction impairs the offspring's ovarian function, such as delayed fetal follicular development in sheep (14), decreased antral follicle numbers in rats and cows (15, 16, 17), and reduced adult progesterone levels later in life in rats (18). The rat offspring of undernourished dams also shows early ovarian aging, characterized by a loss of ovarian follicular reserve and ovarian antioxidant defense impairment in adult rats (15). Furthermore, low birth weight (LBW) causes precocious puberty and age-related abnormalities of estrous cyclicity in rats (18, 19). Thus, a poor nutritional environment during development periods affects reproductive functions later in life; however, its neuroendocrine mechanisms are unknown.

The kisspeptin-GPR54 system has been considered a central reproductive mechanism because the mutations of *Gpr54* (a kisspeptin receptor gene) and *Kiss1* (a kisspeptin gene) in human and rodent models cause puberty failure and reproductive dysfunction in adulthood (20, 21, 22, 23). Kisspeptin neurons in the arcuate nucleus (ARC) coexpress neurokinin B (NKB) and dynorphin A (Dyn), designated as KNDy neurons, and have been considered as an intrinsic gonadotropin-releasing hormone (GnRH)/gonadotropin pulse generator regulating gametogenesis and steroidogenesis in male and female mammals (24, 25, 26, 27). Environmental stresses during development periods such as prenatal androgen excess (28), postnatal estrogen exposure (29, 30), and perinatal overnutrition (31) affect the ARC *Kiss1* expression and/or thereby pulsatile luteinizing hormone (LH) secretion in adulthood. Furthermore, undernutrition during postnatal periods alters pubertal timing due to changes in hypothalamic *Kiss1* expression in female rats (32). However, it is still unclear whether intrauterine malnutrition has long-term adverse effects on ARC KNDy neurons of the female offspring later in life.

The present study investigated the long-term effects of maternal 50% undernutrition on the expression of KNDy genes in female rats at prepuberty and adulthood. Puberty timing, estrous cyclicity, and pulsatile LH secretion were also evaluated to determine if alteration of the KNDy system contributes to the reproductive disorder in prenatally undernourished rats. Additionally, body weight (BW), food intake, accumulation of visceral adiposity, and plasma triglyceride levels as an indicator of metabolic status were assessed.

## Materials and methods

### Animals

**Figure 1:**
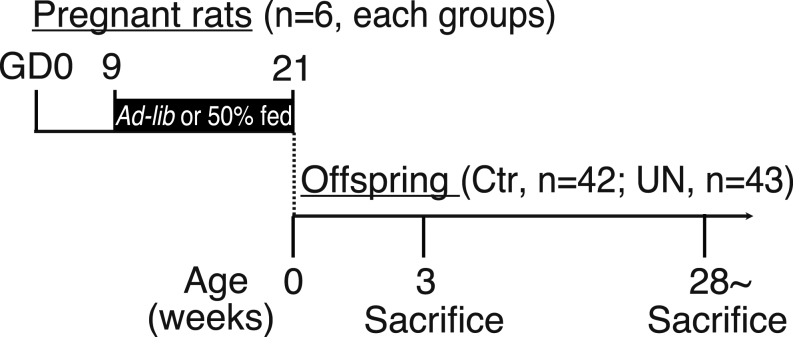
Study schema. Dams were fed either standard chow *ad libitum* (Ctr) or 50% of the daily intake (UN) from gestational day 9 to delivery. All dams were fed *ad libitum* throughout lactation. At weaning (postnatal day 21), part of the Ctr and UN offspring was sacrificed for hypothalamic KNDy gene expression, and the other rats were placed on a normal diet fed *ad libitum* until the animals were sacrificed.

Twelve pregnant, 11 weeks old Wistar-Imamichi strain rats at gestation day (GD) 5 were purchased from Institute for Animal Reproduction (Kasumigaura, Japan). They were housed individually in the facilities at the Nippon Medical School in a controlled environment (14 h light:10 h darkness, lights on at 05:00 h, at 22 ± 2°C) with free access to food and water. The fetal malnutrition model in this study used a modified protocol reported by Slaboda *et al.* in 2009 (18). The pregnant rats were divided into *ad-lib-*fed (control, *n*  = 6) and 50% undernutrition (UN, *n*  = 6) groups. Based on the preliminary experiment for measuring the control group's food intake during gestation, the UN group was fed 50% of the control groups, daily 4 g/100 g BW, during the feeding restriction. Considering that implantation of a fertilized ovum is completed by GD8 in rats (33), restriction feeding for the UN dams was started on GD9. They were restricted to 50% daily food intake of the control dams from GD9 until term delivery. The pups' delivery day was designated day 0 postpartum. BW and sex ratio of pups and litter size were checked, and the litter size was adjusted to eight pups on postnatal day (PND) 1 (Table 1). Upon litter adjustment, female neonates were retained for further analysis. In total, there were 42 female neonates in the control group and 43 in the UN group, and they were weighed weekly until weaning. The weight of neonates was first measured at PND1 because dams are highly sensitive to environmental stimuli following delivery. Of these, some females (*n*  = 7–8) were randomly selected for brain sampling at weaning at PND21. Other 11 female rats from each of the control and UN groups were randomly housed with two or three animals per cage and placed on a normal diet (catalog D12450H; Research Diets, New Brunswick, NJ, USA) for the duration of the study until the animals were sacrificed. The experimental design is provided in Fig. 1A. Since 1 of the 11 animals in both groups eventually died, statistical analysis was performed on the 10 animals for each group for which all BW and estrous cycle data were available. Of these 10 animals, some animals were randomly selected for LH pulse and histological analysis and measurement of the metabolic parameters.

**Table 1:** Effect of maternal food restriction on maternal body weight (BW), pregnancy period, litter size, sex ratio, and birth weight of offspring.

		Ctr	UN
Mother	*n*	6	6
	Maternal BW at gestational day 9	271.02 ± 7.93	274.17 ± 7.70
	Maternal BW at gestational day 21	408.93 ± 12.89	315.63 ± 8.28*
	Maternal BW changes during gestational days (%)^a^	151.1 ± 2.0	113.5 ± 1.0*
	Pregnancy period (day)	22	22
	Litter size	15.83 ± 0.91	15.50 ± 0.76
Offspring	*n* ^b^	95	93
	% of female rats	44.84 ± 2.72	45.95 ± 6.48
	Birth weight of female rats (g)	5.6 ± 0.1	5.2 ± 0.1*

Data are represented as mean ± s.e.m. from Ctr or UN rats.

^a^Maternal BW changes showed the ratio of the BW at gestational day 9 to day 21.

^b^Total offspring number on postnatal day 1 before litter adjustment.

*P < 0.05 compared with Ctr group (Student's t test).

Ctr, normal nutrition control rats; UN, undernutrition rats.

Surgical procedures for the animals were performed under isoflurane anesthesia. The animals were euthanized under deep anesthesia with sodium pentobarbital (100 mg/kg, intraperitoneally (i.p.)) and medetomidine hydrochloride (0.5 mg/kg, i.p.) to harvest brain tissues. The Nippon Medical School’s committee on animal research approved all the procedures and housing conditions used in the study.

### Assessment of maternal undernutrition on reproductive and metabolic parameters in female offspring

The UN (*n*  = 8) or control female offspring (*n*  = 7) at the time of weaning were sacrificed by decapitation to examine hypothalamic KNDy gene expression in prepubertal rats. After removing the entire brain, the hypothalamic tissue, including the ARC and lateral hypothalamic area, was dissected out using a microknife from the coronal sections of the brains sliced with a brain slicer. The coordinates of the brain atlas (34) were 2.28 mm anterior to and 4.44 mm posterior to the bregma. Thereafter, the brain tissue was immediately immersed into ISOGEN (Nippon Gene, Toyama, Japan), homogenized, and stored at −25°C until used for quantitative PCR. The vaginal opening (VO) and first estrus were examined as pubertal signs onset in perinatally undernourished (*n*  = 11) or control female rats (*n*  = 11). Vaginal smears were checked from PND23 to PND70 days to examine the first vaginal estrous timing. The first day when the major cell population in the vaginal smear was cornified cells was designated as the day of the first vaginal estrous. After puberty, vaginal smears were examined daily for 2 weeks at 11, 16, 21, and 26 weeks of age to monitor estrous cyclicity. The length of estrous cycles was defined with the virginal smear date from the start of the estrus to the start of the next estrus. The average length of the observed estrous cycles over 2 weeks was calculated. Food consumption was monitored in some animals at 28–30 weeks of age before surgery. For assessment of pulsatile LH secretion and ARC *Kiss1* and *Tac3* gene expression, perinatally undernourished females (*n*  = 8) or control females (*n*  = 7) were bilaterally ovariectomized (OVX) to remove the influences of endogenous gonadal steroids at 30–41 weeks of age and received a s.c. silastic tubing (1.5 mm inner diameter; 3.0 mm outer diameter; 25.0 mm in length; Dow Corning, Midland, MI, USA) containing estradiol-17β (E2) (Sigma-Aldrich) dissolved in sesame oil at 20 μg/mL 1 week before blood sampling. A previous study demonstrated that rats subjected to this E2 treatment had diestrus plasma E2 levels (35). Blood samples were collected for 3 h at 6-min intervals from freely moving conscious rats from 13:00 h to detect pulsatile LH release. Blood samples (100 μL) were taken from the right atrial cannula (0.5 mm inner diameter and 1.0 mm outer diameter, Shin-Etsu Polymer, Tokyo, Japan) inserted through the jugular vein on the previous day. Each blood sample was replaced with an equivalent amount of washed red blood cells obtained from other rats of same strain to maintain the hematocrit constant. Plasma samples were obtained by immediate centrifugation and stored at −25°C until LH assay. On the next day, the blood (500 μL) for plasma triglyceride assay was additionally collected from the right atrial cannula under deep anesthesia with sodium pentobarbital, and visceral white adipose tissues (retroperitoneal, perirenal, and perigonadal adipose tissues) were weighted. The animals were then perfused with 4% paraformaldehyde (PFA) for histological analysis of the brain.

### Quantitative real-time PCR (qPCR) to examine the hypothalamic KNDy gene expression of female offspring in the prepubertal period

Total RNA was extracted from the hypothalamic tissue of prepubertal rats using ISOGEN according to the manufacturer’s protocols. cDNA was synthesized with oligo dT primers at 50°C using the SuperScript III first-strand synthesis for real-time PCR (Invitrogen). qPCR was performed using fluorescent SYBR green (TB GreenTM Premix Ex TaqTM II, Takara Bio Inc.) and a Thermal Cycler Dice Real-Time System II (Takara Bio Inc.) according to the manufacturer’s instructions. The PCR conditions were as follows: initial denaturation at 95°C for 30 s, followed by 40 cycles of 5 s of denaturation at 95°C, and 30 s of annealing, and extension at 60°C. The oligonucleotide primer sequences and GenBank accession numbers used for qPCR are listed in Table 2. β-Actin (*Actb*) was used as the internal control. Dissociation curve analysis was also performed to ensure the specificity of the PCR.

**Table 2:** Primer set sequences for real-time PCR used in this study.

Gene	Forward primer (5′ to 3′)	Reverse primer (5′ to 3′)	GeneBank accession ID
*Actb*	TGTCACCAACTGGGACGATA	GGGGTGTTGAAGGTCTTCAAA	NM_031144
*Kiss1*	ATGATCTCGCTGGCTTCTTGG	GGTTCACCACAGGTGCCATTTT	XM_017598697
*Tac3*	ATAGGCCAGCAGTGCAGAAA	AGCCAACAGGAGGACCTTG	NM_019162
*Pdyn*	CCTGTCCTTGTGTTCCCTGT	AGAGGCAGTCAGGGTGAGAA	NM_019374

### KNDy gene *in situ* hybridization and kisspeptin immunohistochemistry of female offspring in the adult period

The UN (*n*  = 8) or control (*n*  = 7) female offspring were deeply anesthetized with sodium pentobarbital and perfused with 0.05 M PBS followed by 4% PFA under deep anesthesia with sodium pentobarbital. Brains were harvested immediately, postfixed in the same fixative overnight at 4°C, and then immersed in 20% sucrose in 0.05 M PBS at 4°C. The serial coronal sections (50 μm in thickness) were obtained using a cryostat for histological analysis. Every fourth section through the ARC (from 1.72 to 4.36 mm posterior to the bregma) was obtained from each rat according to the brain atlas (34) for *Kiss1*,* Tac3,* and *Pdyn* *in situ* hybridization (ISH) and kisspeptin immunohistochemistry (IHC). Each series of sections for ISH and IHC contained 12–15 ARC sections, confirming no significant difference in the number of brain sections between the groups. IHC and ISH were performed several times using antibodies and antisense probes whose specificity had been previously confirmed as described later, and the brain sections of both groups were stained in each experiment.

*Kiss1*, *Tac3,* and *Pdyn* mRNA expression were detected by free-floating ISH using specific digoxigenin (DIG)-labeled probes, as described elsewhere (36, 37). DIG-labeled antisense cRNA probes for rat *Kiss1* (position 39–527; GenBank accession no. XM_017598697), *Tac3* (position 180–483, GenBank accession no. NM_019162), and *Pdyn* (position 315–731, GenBank accession no. NM_019374) were synthesized by *in vitro* transcription from the rat hypothalamic cDNA using a DIG-labeling kit (Boehringer Mannheim GmbH, Mannheim, Germany). The specificity of the probes has been confirmed in the past by the fact that no positive signal for the *Kiss1,* *Tac3*, and *Pdyn* mRNA was detected in the brain sections hybridized with the corresponding sense probe (38). Briefly, the brain sections treated with 1 μg/mL proteinase K and 0.25% acetic anhydride in 0.1 M triethanolamine were hybridized with DIG-labeled probes. After hybridization, the sections were treated with 20 μg/mL RNase A (Roche Diagnostics), immersed in 1.5% blocking reagent solution (PerkinElmer), and then incubated with an alkaline phosphatase-conjugated anti-DIG antibody (1:1000, Roche Diagnostics, RRID: AB_514497). The sections were treated with 4-nitroblue tetrazolium chloride/5-bromo-4-chloro-3-indolyl phosphate solution (Roche Diagnostics) until a visible signal was detected. The sections were mounted and examined using an optical microscope (BX50; Olympus) after processing.

According to the method described in our recent report (39), free-floating IHC was performed to detect KISS1 (a kisspeptin protein)-immunoreactive cells. The specificity of KISS1 antibody has been previously demonstrated elsewhere (40). Every fourth section through the ARC was incubated with an anti-KISS1 mouse monoclonal antibody (1:50,000, Takeda #254, kindly donated by Takeda Pharmaceutical Co., Osaka, Japan, RRID: AB_2636957) overnight at 4°C, and the sections were treated with a biotin-conjugated secondary antibody (1:1 in PBST, Histofine SAB-PO kit, Nichirei Biosciences, Tokyo, Japan) for 2 h and then with horseradish peroxidase-conjugated streptavidin for 2 h the next day. Finally, the sections were stained with 3,3′-diaminobenzidine tetrahydrochloride (0.5 μg/mL, Sigma-Aldrich) with 0.03% H_2_O_2_ and then washed with distilled water thrice to stop the reaction. The bright-field images of the sections were obtained with an optical microscope (BX50; Olympus).

### Assays for LH and triglyceride levels

Plasma LH concentrations were determined using a double-antibody RIA with a rat LH RIA kit provided by the National Hormone and Pituitary Program (Baltimore, MD, USA) and were expressed in terms of NIDDK-rLH-RP-3. The least detectable LH level was 0.156 ng/mL for the 50 μL plasma samples. The intra- and inter-assay coefficients of variation were 7.4 and 12.6% at 0.63 ng/mL for 50 μL plasma, respectively. Plasma triglyceride concentrations were determined using a commercial kit (Triglyceride E-test Wako; FUJIFILM Wako Pure Chemical Co.) following kit instructions.

### Data analysis and statistics

Statistical differences in BW changes, the percentage of proestrus, estrus, and diestrus during the estrous cycle, and length of estrous cycles between two groups were determined by two-way ANOVA with maternal diet and time as main effects, followed by the Bonferroni test. Every fourth section through the ARC was counted, and one value/animal was used to quantify the number of *Kiss1*-, *Tac3*-, or *Pdyn*-expressing or KISS1-immunoreactive cells. LH pulses were identified using the PULSAR computer program (41). Statistical differences between the two groups in the other results, including maternal BW at GD9 or 21, maternal BW changes, pregnancy period, litter size, sex ratio of offspring, birth weight of offspring, adipose tissue weight of offspring, plasma triglyceride levels, food intake, each LH pulse parameter, and hypothalamic gene expression levels analyzed by qPCR and histological experiments were determined by the Student’s *t*-test.

## Results

### Establishing a model of low birth weight infants by maternal food restriction

**Figure 2:**
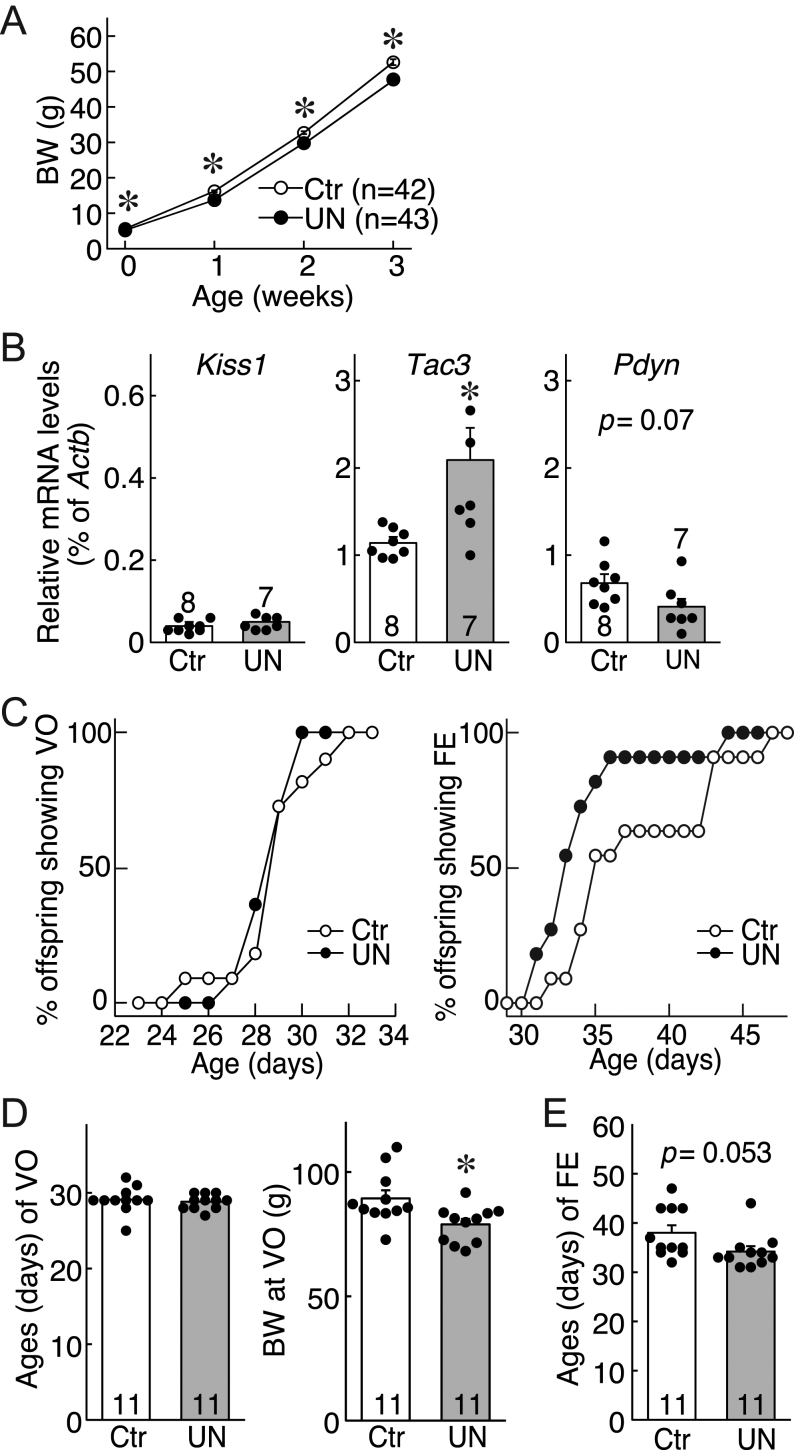
Effect of intrauterine undernutrition on puberty onset of female rats. (A) Changes in body weight (BW) in female offspring of Ctr or UN dams before weaning. Values are mean ± s.e.m. *P < 0.05 (vs Ctr group, two-way). (B) Hypothalamic KNDy gene (*Kiss1*, *Tac3*, and *Pdyn*) expression in Ctr and UN offspring at weaning. The mRNA levels were determined by qPCR. Relative expression levels of each gene were normalized by β-actin gene (*Actb*) expression. (C) Percentage of animals showing vaginal opening (VO) or first estrus (FE). (D) Ages (left) and BW (right) at VO. (E) Ages at virginal FE. *P < 0.05 (vs. Ctr group, Student’s *t*-test). The bar charts portray the mean ± s.e.m. with the individual data points overlaid. The numbers in each column indicate the number of animals used. Ctr, offspring of *ad-libitum*-fed control dams; UN, offspring of undernourished dams.

There were no significant differences in BW between the two maternal groups (control groups and UN groups) on GD9; however, maternal food restriction caused a significant decrease in maternal BW on GD21 (Table 1). The maternal BW changes in the UN group from GD9 to GD21 were significantly lower than those of the control group (*P* < 0.05, Table 1). The BW at PND1 decreased in UN female pups compared to the control because of maternal food restriction (*P* < 0.05, Table 1) but was unaffected by other factors such as the pregnancy period, sex ratio, and litter size (Table 1). The BW in UN pups was significantly lower than that of control from PND1 to 3-week-old rats (*P* < 0.05, Table 1 and Fig. 2A).

### Effect of intrauterine undernutrition on hypothalamic KNDy gene expression in immature female offspring

Prenatal undernutrition results in precocious puberty in female rats (Fig. 2), and UN offspring showed the lower BW until 3 weeks of age, as indicated in Fig. 2A. Figure 2B shows KNDy gene expression in the hypothalamus of prepubertal female rats; UN offspring showed a significant increase in *Tac3* mRNA levels compared with the control (Fig. 2B, middle). UN offspring’s *Pdyn* mRNA levels decreased compared to the control, although the difference was not statistically significant (*P* = 0.07) (Fig. 2B, right). *Kiss1* mRNA levels were comparable between the groups (Fig. 2B, left). A significant difference in the age at VO was not observed between the groups (Figs. 2C and D, left), but the BW at VO was significantly (*P* < 0.05) lower in UN offspring than that in the control (Fig. 2D, right). Fifty-five percent of the UN offspring showed virginal first estrous at 33 days of age, while only 9% of control offspring did at the time (Fig. 2C, right). The first estrous in UN offspring tended to be earlier (*P* = 0.053) than that in the control (Fig. 2E).

### Effect of intrauterine undernutrition on metabolic parameters in mature female offspring

**Figure 3:**
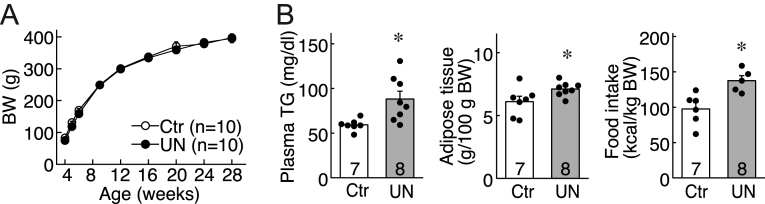
Effect of intrauterine undernutrition on metabolic parameters of female rats. (A) Changes in BW in female offspring of Ctr or UN dams after weaning. Values are mean ± s.e.m. (B) Plasma triglyceride (TG) levels (left), weight of visceral adiposity divided by the BW (middle), and calorie intake (right) in each group. *P < 0.05 (vs Ctr group, Student’s *t*-test). The bar charts portray the mean ± s.e.m. with the individual data points overlaid. The numbers in each column indicate the number of animals used. Ctr, offspring of *ad-libitum* fed control dams; UN, offspring of undernourished dams.

BW, fat accumulation, plasma triglyceride levels, and calorie intake were assessed in mature rats to evaluate whether prenatal undernutrition affects the metabolic parameters of mature offspring. The BW of UN offspring had caught up to that of the control offspring at 4 weeks of age, and thereafter, there were no significant differences in the BW between the two groups (Fig. 3A). However, the UN adult offspring showed a statistically significant (*P* < 0.05) increase in weight of visceral adiposity divided by the BW, plasma triglyceride levels, and calorie intake compared with control offspring in adulthood after 30 weeks of age (Fig. 3B).

### Effect of intrauterine undernutrition on ARC KNDy gene expression in the mature female offspring

**Figure 4:**
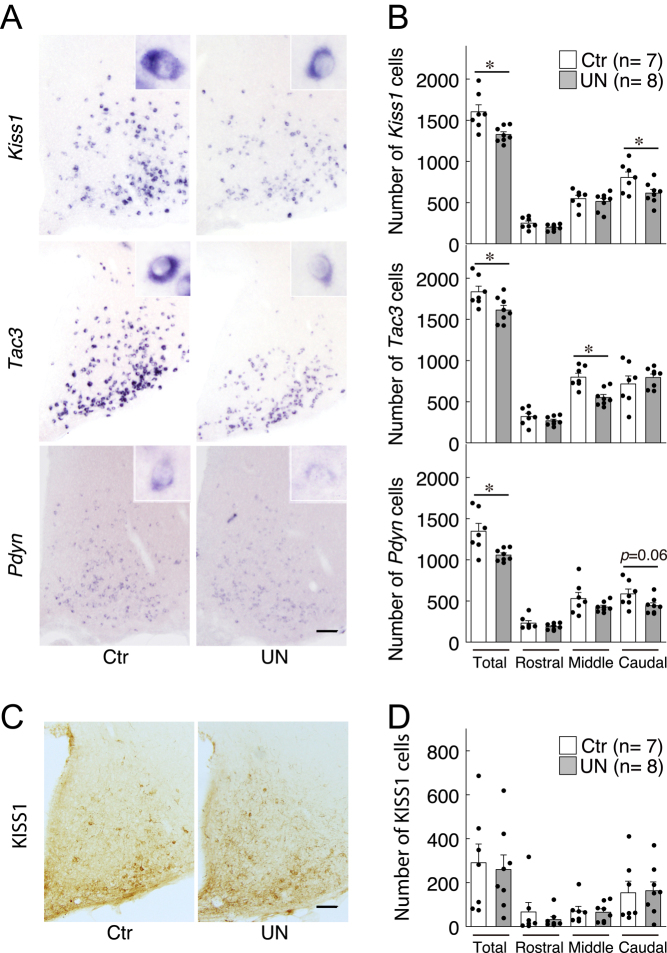
Effect of intrauterine undernutrition on ARC KNDy gene expression in mature female offspring. (A) Representative photomicrographs show *Kiss1*, *Tac3*, and *Pdyn* mRNA distribution using an antisense cRNA probe in the ARC. Scale bar, 100 μm. (B) The number of total *Kiss1*-, *Tac3*-, and *Pdyn*-expressing cells in the whole ARC, or the subtotal number in the rostral, middle, and caudal regions of the ARC. *P < 0.05 (vs the normal-diet controls, Student’s *t*-test). (C) Representative photomicrographs show KISS1-immunoreactive cells in the ARC. Scale bar, 100 μm. (D) The number of total kisspeptin-immunoreactive cells in the whole ARC, or the subtotal number in the rostral, middle, and caudal regions of the ARC. The bar charts portray the mean ± s.e.m. with the individual data points overlaid. Ctr, offspring of *ad-libitum*-fed control dams; UN, offspring of undernourished dams. All animals were OVX and received estradiol a week before the PFA perfusion.

The ARC KNDy gene expression was identified in the E2-primed OVX mature offspring (Figs. 4A and B). The total numbers of ARC *Kiss1*-, *Tac3*-, and *Pdyn* *-*expressing cells in the UN offspring significantly decreased compared to those in the control rats (Fig. 4B). Further analysis of the ARC in three subregions (rostral, middle, and caudal) revealed significant differences in the number of *Kiss1-*expressing cells in the caudal ARC and *Tac3*-expression cells in the middle ARC and also a tendency to decrease in *Pdyn*-expression cells in the caudal ARC between the control and UN groups. The numbers of ARC KISS1-immunoreactive cells in the UN offspring were comparable with that in the control rats (Figs. 4C and D).

### Estrous cyclicity and pulsatile LH secretion in mature UN female offspring

**Figure 5:**
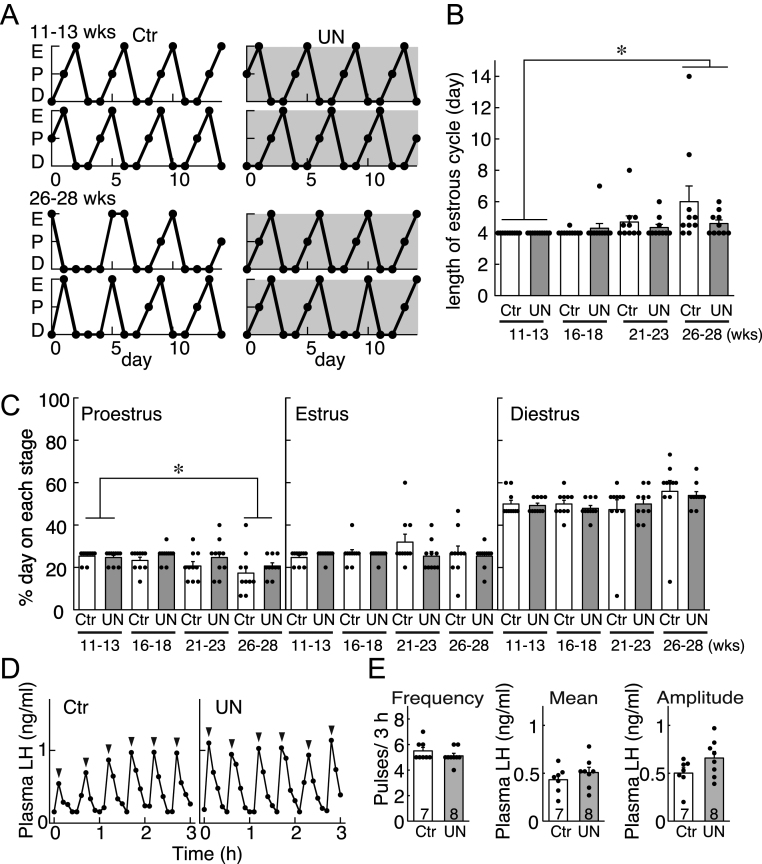
Effect of intrauterine undernutrition on reproductive functions in mature female offspring. (A) Estrous cyclicity in the representative rats in each group at 11–13 and 26–28 weeks of age. Note that the vaginal smears were examined daily for 2 weeks. E, estrus; P, proestrus; D, diestrus. (B) The average length of the estrous cycle in each group (Ctr, *n*  = 10; UN, *n*  = 10) at 11–13, 16–18, 21–23, and 26–28 weeks. (C) The percentages of proestrus, estrus, and diestrus during 2 weeks in each group (Ctr, *n*  = 10; UN, *n*  = 10) at 11–13, 16–18, 21–23, and 26–28 weeks. (D) Plasma LH profiles in the representative animals from each group. All animals were OVX and received estradiol. After 1 week, blood samples were collected every 6 min for 3 h. Arrowheads indicate the peaks of the LH pulses as identified using the PULSAR computer program. (E) Frequency of the LH pulses, mean LH concentration, and amplitude of the LH pulses were calculated for a 3 h sampling period. *P < 0.05 (vs 11–13 weeks, two-way ANOVA followed by the Bonferroni test). The bar charts portray the mean ± s.e.m. with the individual data points overlaid. The numbers in each column indicate the number of animals used. Ctr, offspring of* ad-libitum*-fed control dams; UN, offspring of undernourished dams.

Vaginal smear was analyzed for 2 weeks and at 11–13, 16–18, 21–23, and 26–28 weeks of age to determine the prenatal undernutrition effect on estrous cyclicity, which was disrupted with aging in both UN and control offspring. From 11 to 13 weeks of age, all offspring in both groups showed a regular 4-day estrous cycle (>3 cycles/2 weeks) (Figs. 5A and B). Similarly, almost all UN and control offspring showed a regular 4- or 5-day estrous cycle at 16–18 and 21–23 weeks of age (Fig. 5B). At 26–28 weeks of age, both UN and control offspring showed age-related changes in estrous cyclicity, such as unclear proestrus, extended diestrus, or extended estrus (Fig. 5A lower and 5C). Estrous cycle length showed no significant interaction between maternal diet and age; however, the age effect was significant, and the cycles of 26–28 weeks were significantly longer than those of 11–13 weeks (Fig. 5B).

As for the profiles of LH release in the mature E2-primed OVX models, apparent LH pulses were found in both groups at 30–41 weeks of age (Fig. 5C). Prenatal undernutrition did not affect the mean LH levels and LH pulse amplitude and frequency of mature offspring (Fig. 5D).

## Discussion

This study showed that prenatal undernutrition results in prepubertal increase in hypothalamic NKB gene (*Tac3*) expression, followed by a tendency to advance first virginal estrus. Conversely, UN significantly suppressed ARC KNDy gene expression in middle-aged rats after 30 weeks of age, suggesting that intrauterine undernutrition stress results in developmental programming of KNDy neurons, which changes in their gene expression depending on life stages. However, mature UN offspring showed normal estrous cycle and LH pulses similar to control offspring. Thus, a GnRH/LH pulse generator is a robust mechanism to maintain normal reproductive function against deterioration of the uterine nutritional environment. Additionally, prenatal undernutrition stimulates obesogenic effects, such as rapid catch-up growth at 4 weeks following LBW, visceral fat accumulation, and increased calorie intake and plasma triglyceride levels in adulthood. These metabolic symptoms agree with the thrifty phenotype hypothesis that an adverse intrauterine environment such as caloric restriction induces developmental programming of the fetal hypothalamus and peripheral organs, altering the fetal metabolic and hormonal milieu, predisposing to metabolic syndrome (42). The current study collectively demonstrated that intrauterine undernutrition caused hypothalamus developmental programming resulting in central precocious puberty and obesogenic profile, but not associated with GnRH/LH pulses at adulthood in female rats.

The prenatal-undernutrition-induced advanced first vaginal estrous may have occurred due to changes in prepubertal hypothalamic *Tac3* gene expression since these genes have a stimulatory role in pulsatile GnRH/LH secretion (24). Previous pharmacological studies showed that administration of an NKB receptor agonist or a dynorphin receptor antagonist accelerated puberty onset in female rats (43, 44). Thus, intrauterine undernutrition might cause a prepubertal increase in *Tac3* gene expression in KNDy neurons to accelerate puberty onset through pulsatile GnRH/LH secretion in the UN female offspring. The present fetal undernutrition showed a tendency toward a decrease in prepubertal hypothalamic *Pdyn* gene expression, which may also contribute to accelerating pulsatile GnRH/LH secretion.

Nutritional or energy status are important determinants of pubertal initiation, as indicated by delayed puberty due to food restriction and advanced puberty due to overnutrition in mammals (32, 45). Prepubertal female rats fed a high-fat diet induced rapid BW growth and then a profound advancement of puberty concomitant with a dramatic acceleration of LH pulse frequency that is paralleled by a significantly earlier dynamic rise in ARC *Tac3* expression (45). It is suggested that ARC *Tac3* is a central reproductive regulator of the timing of puberty in response to peripubertal metabolic changes. It is tempting to speculate that the peripubertal rapid catch-up growth of the UN rats in the current study may underlie the concomitant increase in hypothalamic *Tac3* expression and then pubertal acceleration in the UN rats. Indeed, the previous study demonstrated that the UN offspring showed prepuberal obesogenic effects such as elevated leptin, rapid weight gain, and increased adipose tissue mass despite the low birth weight (46). Thus, the metabolic changes in the UN rats might alter hypothalamic *Tac3* expression, resulting in the advanced first virginal estrus.

The present study showed the discrepancy between the decrease in the number of ARC KNDy neurons and unchanged LH pulses by fetal malnutrition, indicating the possibility of a compensatory mechanism against the reduction of ARC *Kiss1* expression to maintain a regular pulsatile GnRH/LH secretion. Several mice models with ARC *Kiss1* knockdown and global *Kiss1* KO have also shown that a compensatory mechanism may rescue reproductive functions (47, 48). The present result showed an UN-induced reduction of cell number expressing *Kiss1* or *Tac3* in a different ARC subdivision, suggesting that it could be attributed to a suppression of KNDy gene expression in adulthood rather than cell death in the KNDy neurons during developmental periods. The notion is supported by the present result that UN animals showed comparable prepubertal hypothalamic *Kiss1* levels and ARC KISS1 immunoreactivity to control groups. The normal ARC KISS1 immunoreactivity in the fetal UN likely implies that the peptide secretion from the ARC was also unchanged, resulting in regular GnRH/LH secretion in the UN rats. It should be noted that GnRH may also have a compensatory role in maintaining normal GnRH/LH pulses in the UN female rats, following a previous report that fetal UN caused increased hypothalamic *Gnrh1* gene expression in 10-month-old female rats (19).

Our results showed accelerated sexual maturation that resulted from intrauterine undernutrition. Sloboda’s study also indicated that puberty was advanced by fetal undernutrition in female rat offspring born from mothers with 50% food restriction throughout gestation and/or lactation; the authors discussed their results based on life-history theory (18). The organism trades length of life for reproduction in a threatening circumstance; therefore, poor nutrition or threatening circumstances in early life leads to precocity (49). The theory is supported by clinical observations; LBW followed by accelerated postnatal growth has been associated with earlier menarche in humans (50, 51, 52, 53). Besides the accelerated sexual maturation, fetal undernutrition also resulted in ovulation disorders and low blood estrogen levels in middle age, which were associated with premature reproductive aging in female rats (19). These studies were also supported by our results showing a reduction of ARC KNDy gene expression in middle-aged UN offspring, but a limitation of the present study is that the long-term effects of the gene expression reductions on reproductive functions could not be examined. At the time of KNDy gene expression analysis in this study (i.e. 26–28 weeks of age), the sexual cycle was prolonged in both groups compared to that at 11–13 weeks. However, most animals exhibited a 4–5-day estrous cycle, suggesting that they were in the early stages of aging in the present study. Further studies with older UN animals than 29 weeks of age will be required to clarify this point.

The period of early gestation may be important for fetal hypothalamic programming in fetal UN-induced advanced puberty because maternal food restriction from GD9 to delivery tends to advance puberty in the offspring. Conversely, female rat offspring of restricted maternal food from GD14 to delivery showed delayed puberty due to suppression of prepubertal hypothalamic *Kiss1* expression (32). The discrepancy between these results could be attributed to the difference in the maternal undernutrition period. Hence, hypothalamic fetal programming related to precocious puberty may require prolonged undernutrition in the fetus or the experience of nutritional deficiency by GD13.

This study demonstrated that intrauterine undernutrition affected hypothalamic developmental programming in female rats, which induced long-term effects on hypothalamic KNDy gene expression and could influence reproductive functions later in life. Intrauterine undernutrition induced rapid catch-up growth and metabolic changes during peripubertal periods that caused central precocious puberty by altering expression in hypothalamic KNDy gene. Furthermore, the fetal UN-induced decrease in ARC KNDy gene expression in middle-aged rats suggests that the fetal UN may accelerate the age-related decline in KNDy gene expression, eventually leading to central premature reproductive senescence in mature female rat offspring. In the preventive medicine context, this study provides evidence that maternal nutritional management is essential for offspring’s reproductive health and would be useful for medical treatment for reproductive disruption in patients with intrauterine growth retardation.

## Declaration of interest

The authors declare no conflict of interest.

## Funding

This work was supported in part by the Grants-in-Aid from the Japan Society for the Promotion of Science http://dx.doi.org/10.13039/501100001691 (grant numbers 20J40270, 20K16123, and 18K06860); the Nippon Medical School Grant-in-Aid for Young and Women Investigators; the Initiative for Realizing Diversity in the Research Environment from Ministry of Educationhttp://dx.doi.org/10.13039/100010002, Culture, Sports, Science and Technology (MEXT); and the MEXT-supported Program for Strategic Research Foundation at Private Universities.

## Data availability statement

The data that support the findings of this study are available from the corresponding author upon reasonable request.
